# Immunosenescence and Immune Exhaustion Are Associated with Levels of Protein-Bound Uremic Toxins in Patients on Hemodialysis

**DOI:** 10.3390/biomedicines11092504

**Published:** 2023-09-11

**Authors:** Theodoros Tourountzis, Georgios Lioulios, Steven Van Laecke, Evdoxia Ginikopoulou, Vasiliki Nikolaidou, Eleni Moysidou, Stamatia Stai, Michalis Christodoulou, Asimina Fylaktou, Griet Glorieux, Maria Stangou

**Affiliations:** 1 Protypo Dialysis Center of Thessaloniki, 55535 Thessaloniki, Greece; ginikopoulou@gmail.com; 2 Department of Nephrology, General Hospital “Hippokratio”, School of Medicine, Aristotle University of Thessaloniki, 54642 Thessaloniki, Greece; pter43@yahoo.gr (G.L.); moysidoueleni@yahoo.com (E.M.); staimatina@yahoo.gr (S.S.); michalischristodoulou22@gmail.com (M.C.); mstangou@auth.gr (M.S.); 3 Department of Internal Medicine and Pediatrics, Nephrology Unit, Ghent University Hospital, 9000 Gent, Belgium; steven.vanlaecke@ugent.be (S.V.L.); griet.glorieux@ugent.be (G.G.); 4 Department of Immunology, National Peripheral Histocompatibility Center, General Hospital “Hippokratio”, 54642 Thessaloniki, Greece; basoniko@hotmail.com (V.N.); fylaktoumina@gmail.com (A.F.)

**Keywords:** protein-bound uremic toxins, immunosenescence, immunoexhaustion, hemodialysis, kidney failure

## Abstract

Background: The accumulation of protein-bound uremic toxins (PBUTs) in chronic kidney disease may affect patients’ immune status. The aim of the study was to evaluate their potential impacts on lymphocyte alterations in patients on hemodialysis (HD). Methods: The plasma levels of PBUTs were assessed in 54 patients on HD and 31 healthy individuals, using ultra-performance liquid chromatography. The results correlated with the senescent and exhausted status of lymphocytes, based on certain surface molecules, analyzed by flow cytometry. Results: The plasma levels of PBUTs were significantly increased in the patients on HD compared with the healthy controls. The patients with residual kidney function had reduced hippuric acid (HA) levels, total (*p* = 0.03) and free (*p* = 0.04), and free IxS levels (*p* = 0.02). The total and free HA levels correlated negatively with less differentiated subpopulations, CD4+CD45RA+CD31+ (*p* = 0.037 and *p* = 0.027), CD8+CD28+CD57− (*p* = 0.01, *p* = 0.01), and naïve B cells (CD19+IgD+CD27−) (*p* = 0.04, *p* = 0.03). Both the total and the free pCS levels correlated positively with exhausted CD4 cells, *p* = 0.02 and *p* = 0.01, respectively. A multivariate analysis showed that IxS and age were the main independent parameters implicated in the reduction intotal CD4 and B lymphocytes and their naïve and early differentiated subsets. Conclusions: Increased PBUTs levels are associated with immune disturbances of patients on HD, HA, and IxS in the immunosenescent and pCS in the immunoexhaustion alterations.

## 1. Introduction

The adaptive immune system is principally affected by chronic kidney disease (CKD). Alterations encompass total T and B cell lymphopenia, involving a reduction in naïve and early differentiated lymphocyte populations and the simultaneous accumulation of advanced differentiated subsets. Immunosenescence and immunoexhaustion have been described in the presence of CKD starting at early stages and deteriorate gradually, toward kidney failure (KF) and initiation of hemodialysis (HD) [1,2]. Multiple factors related to impaired kidney function may be implicated. Gut dysbiosis expressed as alterations in the gut microbiome, frequently seen in kidney disease, can stimulate immune system alterations [3]. The disturbance of the physiological gut microbiota is implicated for intestinal dysbiosis, impairment of the function of the intestinal barrier, and translocation of bacteria. As a result of the intestinal microbiome alteration, excessive uremic toxins are produced, which will leak into the circulation, along with endotoxin and microorganism fragments [4]. This endotoxemia conduces to a condition of low-grade chronic inflammation and oxidative stress, leading to additional decline of renal function. Thus, a vicious cycle between the kidney and the intestine is generated [5].

The European Uremic Toxin Work Group (EUTox), in 2003, categorized uremic toxins in three groups, according to their physicochemical characteristics: free water-soluble low-molecular-weight solutes (lower than 500 Da), protein-bound solutes, and middle-molecular-weight molecules (above 500 Da) [6]. The classification of especially the latter molecules was more recently updated [7]. Protein-bound uremic toxins (PBUT) have low molecular weight [indoxyl sulfate (IxS): 212 Da; indole-3-acetic acid (IAA): 175 Da; p-cresyl sulfate (pCS): 31 Da; hippuric acid (HA): 179 Da; p-cresyl glucuronide (pCG): 284 Da and 3-carboxy-4-methyl-propyl-2-furanpropanoic acid (CMPF): 240 Da], but they are for the largest part bound to protein [8,9]. As a result, only the free fraction can be removed by using current standard HD methods and through conventional membranes, explaining why residual kidney function (RKF) could significantly modify their plasma concentration in patients on dialysis [10]. PBUT retention solutes accumulate in patients with KF, resulting in increased toxicity, with detrimental clinical effects, including high morbidity and mortality [7]. The accumulation of PBUTs also promotes dysfunction of the innate and adaptive immune system: toxicity on endothelial cells, chronic activation of neutrophils and monocytes, impaired antibacterial capacity and antigen presentation, and defective cellular and humoral immunity based on the dysfunction of T and B lymphocytes [11]. Whether these effects on lymphocyte function contribute to the advanced senescent and exhausted phenotype of patients with KF has not been investigated yet.

Certain characteristics of immunosenescence, such as premature thymic involution and reduced thymic output, the elimination of early differentiated T lymphocytes, and down-regulation of the CD28 co-stimulatory receptor, have already been described, in the presence of kidney impairment [12,13].

The accumulation of PBUTs is likely to participate in immunosenescent and immunoexhausted changes seen in KF patients; however, no previous studies have investigated this hypothesis. The purpose of the present study was to evaluate the potential association of PBUT accumulation with aging-associated T cell changes in patients with KF undergoing HD.

## 2. Materials and Methods

### 2.1. Study Population

Fifty-four Caucasian patients with KF, treated with HD, and 31 healthy individuals of similar age, sex, and ethnicity, serving as controls, were included in the present cross-sectional study. The control group consisted of healthy individuals, working in the hospital or dialysis unit.

#### Patient Inclusion and Exclusion Criteria

Patients with KF were eligible to participate if they fulfilled the following criteria: age above 18 years, kidney replacement treatment with HD for at least 1 year, and under stable conditions of the dialysis method.

The exclusion criteria were the presence or history of diabetes mellitus, recent infection or vaccination (last 3 months), abnormal C-reactive protein (CRP), autoimmune disease or immunosuppressive treatment during the last 12 months, and active malignancy or a history of malignancy during the last 5 years.

RKF was defined as the presence of urine output ≥ 500 mL/24 h, and patients on HD were further divided as those with or without RKF.

### 2.2. Laboratory Methods

Blood samples were collected, before the initiation of a mid-week dialysis session, in Ethylene diamine tetraacetic acid (EDTA) tubes; part was used to analyze the total lymphocyte count and their subsets by flow cytometry, and part was centrifuged for 10 min at 2095× *g* to collect plasma, which was stored at −60 °C until a batch analysis by using ultra-performance liquid chromatography (UPLC) was conducted to quantify the uremic toxin levels.

Additional data that were recorded as laboratory parameters were as follows: complete blood count, serum urea (in mg/dL, enzymatic method), serum creatinine (in mg/dL, enzymatic kinetic method), serum calcium (in mg/dL, colorimetric method), serum phosphorus (in mg/dL, colorimetric method), parathyroid hormone (in pg/mL, chemiluminescent microparticle immunoassay method), serum cholesterol (in mg/dL, enzymatic method), serum triglycerides (in mg/dL, enzymatic method), serum high-density lipoprotein cholesterol (HDL-C, in mg/dL, enzymatic method), serum low-density lipoprotein cholesterol (LDL-C, in mg/dL, enzymatic method), ferritin (in ng/mL, chemiluminescent microparticle immunoassay method), serum albumin (in g/dL, colorimetric method), serum lactate dehydrogenase (LDH, IU/L, enzymatic kinetic method), and C-reactive protein (CRP, in mg/L, immunostaining method).

#### 2.2.1. Uremic Toxins Analysis

The sample preparation for total toxin concentration was as follows: 100 μL of plasma was initially diluted with 260 μL of UPLC grade water (Thermo Scientific, Geel, Belgium). For heat deproteinization, the samples were placed at 95 °C for 30 min, cooled in an ice bath for 10 min, and centrifuged at 18,000× *g* for 10 min. The supernatant was centrifuged through a 30 kDa cutoff centrifugal filter (Amicon Ultra 0.5, Merck KGaA, Darmstadt, Germany) for 20 min at 4500× *g*. For the free concentration, 260 μL of untreated plasma was initially centrifuged through a 30 kDa cutoff centrifugal filter at 4500× *g* for 20 min, and 100 μL of the ultrafiltrate was diluted with 260 μL of UPLC-grade water followed by the same heat treatment as described above. Finally, 180 μL of the ultrafiltrate was transferred into a vial, and internal standard (fluorescein; 50 ppm) was added. UPLC (Agilent 1290 Infinity device; Agilent, Santa Clara, CA, USA) was used to separate the uremic toxins. HA and CMPF were detected with an Agilent G4212A diode array detector at 245 nm and 254 nm, respectively. Indoxyl sulfate (λex: 280 nm, λem: 376 nm), p-cresyl sulfate and p-cresyl glucuronide (λex: 264 nm, λem: 290 nm), indole-3-acetic acid (λex: 280 nm, λem: 350 nm), and fluorescein (λex: 443 nm, λem: 512 nm) were detected by an Agilent G1316C fluorescence detector.

#### 2.2.2. Lymphocytes’ Analysis

Flow cytometric analysis was performed within 12 h after collection. Certain lymphocyte surface receptors, representative of their aging and exhaustion (CD45RA, CCR7, CD28, CD57, and PD1 for T lymphocytes and IgD and CD27 for B lymphocytes), were evaluated and followed by further analysis, which determined the lymphocytes’ subsets by using a cell counter (Navios Flow Cytometer, Beckman Coulter, Indianapolis, IN, USA), as described before [13].

The conjugated antibodies used for blood sample staining were anti-CD45 PC7 J33 (IM3548U, Beckman Coulter), anti-CD3 FITC UCHT1 (A07746, Beckman Coulter), anti-CD3 PE UCHT1 (A07747, Beckman Coulter), anti-CD4 Pacific blue MEM-241 (PB-359-T100, EXBIO, Praha SA, Czechia), anti-CD8 PC5 B9.11 (A7758, Beckman Coulter), anti-CD45RA APC MEM-56 (1A-223-T100, EXBIO), anti-CCR7 PE 4B12 (1P-735-C100, EXBIO), anti-CD28 CD28.2 PE-EF610 (61-0289-42, ThermoScientific LSG, Waltham, MA, USA), anti-CD31 APC MEM05 (T5-273-T100, EXBIO), anti-CD57 FITC TB01 (1F-158-T100, EXBIO), anti-CD279 (PD1) EI12.2H7 (11-176-C100, EXBIO), anti-CD19 PC5 J3-119 (Beckman Coulter), anti-IgD IA6-2 (Thermo Scientific LSG), and anti-CD27 PE-DyLight 594 (EXBIO). Fluorescence Minus One (FMO) contrast was used.

CD4 and CD8 T cells were classified as follows:

Early differentiated: recent thymic emigrants (RTEs),CD4+CD45RA+CD31+ and CD8+CD45RA+CD31+; naïve cells, CD4+CD45RA+CCR7+, CD4+CD28+CD57−, CD8+CD45RA+CCR7+, and CD8+CD28+CD57−.

Memory cells: central memory (CM) cells, CD4+CD45RA−CCR7+ and CD8+CD45RA−CCR7+; effector memory (EM) cells, CD4+CD45RA−CCR7− and CD8+CD45RA−CCR7−.

Advanced differentiated, senescent cells: effector memory re-expressing CD45RA cells (EMRA), CD4+CD45RA+CCR7−, CD8+CD45RA+CCR7−, CD4+CD45RA−CD57+, CD8+CD45RA−CD57+, CD4+CD28+CD57+, and CD8+CD28+CD57+.

Terminally differentiated, senescent cells: EMRACD28- (CD4+CD45RA+CCR7−CD28− and CD8+CD45RA+CCR7−CD28−), CD4+CD28−CD57+, and CD8+CD28−CD57+.

Exhausted cells: PD1+, CD4+CD45RA−PD1+, CD4+CD45RA+PD1+, CD8+CD45RA−PD1+, and CD8+CD45RA+PD1+.

B cells were classified as naïve (IgD+CD27−), IgM memory (IgD+CD27+), switched memory (IgD−CD27+), and double negative (IgD−CD27−).

Figures S1 and S2 describe the gating strategies of the T and B lymphocyte subpopulations.

### 2.3. Statistical Analysis

The statistical processing and analysis of the data was performed with the statistical package for social sciences (SPSS) 27 IBM Corp, Armonk, NY, USA, for Windows. The level of statistical significance (*p*) was set below 0.05. The qualitative variables were described using absolute (n) and relative frequency (%). Kolmogorov–Smirnov and Shapiro tests were used to estimate the normality distribution of continuous variables. Normally distributed parameters were expressed as mean ± standard deviation (SD), while non-normally distributed parameters were expressed as the median and range. A Mann–Whitney U test was performed to estimate the differences in parameters between two groups, and a Spearman’s correlation test was used to assess the correlations between non-parametric variables. The Spearman’s coefficient and multiple regression analysis were performed to estimate the correlation between uremic toxin blood levels and lymphocyte subpopulations and to define independent factors for certain subsets.

Informed consent was obtained from all subjects involved in the study. The study was conducted in accordance with the Declaration of Helsinki and approved by the Institutional Review Board (or Ethics Committee) of the Medical School of the Aristotle University of Thessaloniki (protocol code 134/2023). All research activities were performed with coded-pseudonymized tissue samples and data.

## 3. Results

### 3.1. Description of Patient Data

The clinical and demographic characteristics of the 54 patients on HD, 23 females, mean age 51.3 ± 16.9 years, and the 31 healthy individuals, 15 females, mean age 51.3 ± 17.2 years, are depicted in Table 1 and Table 2. The causes of CKD were primary glomerulonephritis in 16/54 (29.6%), obstructive uropathy in 9/54 (16.7%), autosomal dominant polycystic kidney disease in 7/54 (13%), hypertension in 3/54 (5.6%), Alport syndrome in 2/54 (3.7%), other in 2/54 (3.7%), and unknown in 15/54 (27.8%) patients. None of the patients had hepatitis B or C.

### 3.2. Differences in Lymphocytes and Their Subpopulations

A significant increase in the numbers of white blood cells was noticed in the patients on HD compared with the controls (*p* = 0.046), which was entirely attributed to the expansion of neutrophils and monocytes (*p* = 0.001 and *p* = 0.001, respectively), while lymphocytes were significantly reduced (*p* < 0.001) (Table 2). The absolute number of CD4+ lymphocytes per µL was also reduced (*p* < 0.001), with this reduction affecting mainly naïve and less differentiated subpopulations. The changes in cell subsets did not correlate with the Kt/V ratio or other dialysis characteristics (such as the duration, vascular access, or membrane material).

As shown in Table 3, almost all absolute numbers of early differentiated subsets and central memory CD4 cells were reduced. Only a few of the early differentiated CD8 lymphocyte subsets were also decreased, including CD8+CD28+CD57− (Table 3).

The reduction in the B cell compartment included the whole number of CD19+ cells and affected equally all subpopulations (Table 4).

In contrast, the increased expression of the PD1 molecule was found in patients on hemodialysis and mainly affected CD4 cells and particularly CD4+CD45RA-PD1+ cells (Table 5).

### 3.3. Concentration of Protein-Bound Uremic Toxins in Patients on Hemodialysis vs. Control

A higher concentration of uremic toxins was observed in patients on HD compared with the healthy control group, affecting both the total and the free levels of uremic toxins. Specifically, the levels of PBUT in the control group and patients on HD were as follows: total HA, 0.102 (0.04–0.2) and 3.05 (1.66–5.37) mg/dL (*p* < 0.001); free HA, 0.029 (0.03–0.04) and 1.482 (0.7–2.8) mg/dL (*p* < 0.001); total IxS,0.063 (0.04–0.09) and 2.207 (1.27–3.34) mg/dL (*p* < 0.001); free IxS, 0.0004 (0.0004–0.0004) and 0.146 (0.09–0.27) mg/dL (*p* < 0.001); total pCS, 0.066 (0.04–0.13) and 1.248 (0.84–1.66) mg/dL (*p* < 0.001); free pCS, 0.004 (0.004–0.005) and 0.089 (0.06–0.13) mg/dL (*p* < 0.001); total pCG, 0.0017 (0.0013–0.0017) and 0.2243 (0.0935–0.3854) mg/dL (*p* < 0.001); free pCG, 0.0017 (0.0011–0.0017) and 0.1976 (0.0803–0.3503) mg/dL (*p* < 0.001); total IAA, 0.0283 (0.0223–0.0347) and 0.1177 (0.0884–0.1668) mg/dL (*p* < 0.001); free IAA, 0.0063 (0.0056–0.0069) and 0.0403 (0.0291–0.0534) mg/dL (*p* < 0.001); and total CMPF, 0.0716 (0.035–0.1446) and 0.169 (0.0998–0.3767) mg/dL (*p* < 0.001).

### 3.4. Correlation of PBUTs with Clinical Features

**Figure 1:**
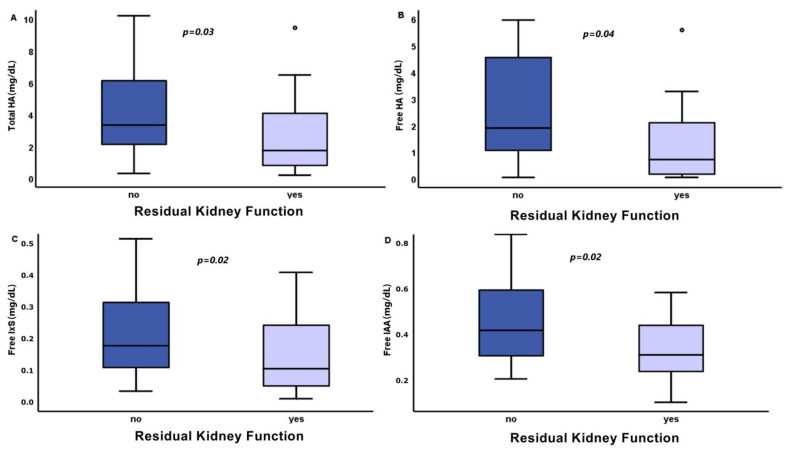
Differences in concentration of total (**A**) and free (**B**) levels of hippuric acid (HA), free levels of indoxyl sulfate (IxS) (**C**), and free indole-3-acetic acid (IAA) levels (**D**) in dialysis patients without (no) and with (yes) residual urine output.

The PBUT levels in the patients on HD did not have statistically significant correlations with the sex, age, prescription, or method of dialysis and dialysis membrane materials. The concentrations of HA (total and free) had a positive correlation with the HD vintage (in months) (r = 0.4, *p* = 0.005 and r = 0.4, *p* = 0.003, respectively). Patients with residual kidney function had reduced total and free HA levels, 1.739 (0.624–4.105) vs. 3.347 (2.107–6.269) mg/dL, *p* = 0.03, and 0.717 (0.158–2.099) vs. 1.901 (1.055–4.598) mg/dL, *p* = 0.04, respectively, and free IxS levels, 0.106 (0.048–0.242) vs. 0.177 (0.108–0.319) mg/dL, *p* = 0.02 (Figure 1). The levels of the other examined uremic toxins did not show correlations with the residual kidney function.

### 3.5. Correlation of PBUTs with the Immunological Profile

The lymphocyte count and absolute number of CD4 cells showed a significant negative correlation with the total (r = −0.3, *p* = 0.01 and r = −0.3, *p* = 0.02, respectively) and free HA levels (r = −0.3, *p* = 0.02 and r = −0.3, *p* = 0.01, respectively). Moreover, several naïve and less differentiated CD4 subpopulations also demonstrated a negative correlation with the total and free HA levels, most importantly, CD4+CD45RA+CD31+ (r = −0.3, *p* = 0.037 and r = −0.3, *p* = 0.027, respectively), CD4+CD45RA+CD57− (r = −0.3, *p* = 0.03 and r = −0.3, *p* = 0.02, respectively), CD4+CD28+CD57− (r = −0.3, *p* = 0.05 and r = −0.3, *p* = 0.03, respectively) (Table 6), and CD8+CD28+CD57− (r = −0.3, *p* = 0.01 and r = −0.3, *p* = 0.01, respectively). The CMPF, IAA, and pCG levels did not show any correlation with either early or late differentiated lymphocytes, and they are not included in the table.

The exhausted CD4 cells had positive relationships with the total and free levels of pCS (r = 0.3, *p* = 0.02 and r = 0.3, *p* = 0.018, respectively) but not with the HA levels. In addition, for further divided exhausted lymphocytes, according to CD45RA expression, CD4+CD45RA+PD1+ had similar relationships with total and free pCS (r = 0.3, *p* = 0.039 and r = 0.3, *p* = 0.045, respectively) (Table 7).

The number of B lymphocytes (CD19+) had a negative correlation with free HA (r = −0.2, *p* = 0.05) and free IxS (r = −0.4, *p* = 0.008) levels. Naïve and non-switched memory B cell subsets (CD19+IgD+CD27− and CD19+IgD+CD27+) showed strong negative associations with HA, IxS, pCG, and CMPF (Table 8).

### 3.6. Independent Parameters Participating to Lymphocyte Subsets

Multiple regression analyses, including age and RKF for all subpopulations examined; free HA and IxS (for early CD4 lymphocyte subsets) or pCS (for exhausted CD4 cells); and free HA, IxS, and free pCG (for B lymphocytes) were performed to assess independent factors contributing to the most important lymphocyte subsets. The included independent factors were the free uremic toxin blood levels, the HD vintage, and the presence of RKF.

**Figure 2:**
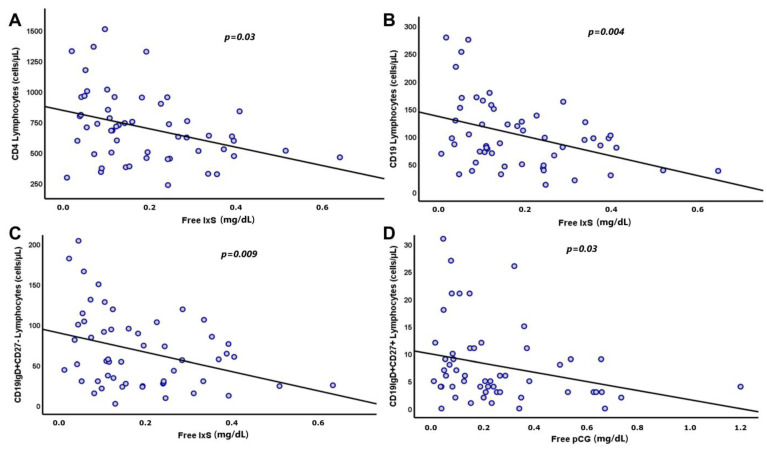
Linear association of T and B cell subsets with free levels of indoxyl sulfate (IxS) and p-cresyl glucuronide (pCG). Blood levels of free IxS had significant negative correlations with total CD4 lymphocytes (**A**), B lymphocytes (**B**), and CD19IgD+CD27− cells (**C**), while blood levels of free pCG had negative correlations with CD19IgD+CD27+ cells (**D**).

The age and uremic toxins levels, for instance, of IxS mainly, had significant and independent roles in the reduction in the total CD4 and B lymphocytes, as well as their early differentiated subtypes (Table 9 and Figure 2).

## 4. Discussion

In the present study, we assessed the potential association between PBUTs and disorders of the adaptive immunity in hemodialysis patients, concentrating primarily on senescent and exhausted phenotypic changes of the peripheral lymphocytes.

As anticipated, increased levels of all evaluated PBUTs, including HA, IxS, pCS, IAA, pCG, and CMPF, were observed in our patients on hemodialysis compared with the healthy controls, a finding apparently attributed to their compromised removal by dialysis membranes [14,15]. A recent study has described immunosenescent and immunoexhaustion phenotypic changes in the presence of KF [13]. Data demonstrating a relationship between this profile and certain disorders in KF are currently lacking. Because the accumulation of PBUTs in patients on hemodialysis is expected to have an important implication on the immune status, we decided to estimate the potential association of PBUT levels with lymphocyte alterations.

Patients included in the study, treated with HD for at least one year, were found to have a significantly increased white blood cell and neutrophil count, however, with a lower number of lymphocytes. The reduction in lymphocytes affected mainly CD4+ and B cells. More specifically, most impaired CD4 subpopulations were naïve (CD4+CD45RA+CD31+, CD4+CD45RA+CD57−, CD4+CD28+CD57−, and CD8+CD28+CD57−) and memory (CD4+CD45RA−CCR7+) cells, while B lymphocytes were evenly reduced, with their subpopulations retaining similar proportions to those of the healthy control group. Nevertheless, terminally differentiated (CD4+CD28−CD57+ and CD4+CD45RA+CCR7−CD28−) and exhausted CD4 lymphocytes (CD4+PD1+ and CD4+CD45RA−PD1+) were increased in the patient group. Similar changes, leading to a shift toward a senescent and/or exhausted subtype, was described in CD8 lymphocytes; however, the changes were less prominent.

Total and free HA and IxS levels were negatively correlated with the total lymphocyte count, absolute number of CD4 cells, and, very interestingly, almost all naïve and less differentiated CD4 and B lymphocyte subpopulations. Instead, the concentrations of pCS did not show any association with markers of senescence, but they had positive correlations with the exhausted T cell subpopulations, such as CD4+PD1+ and CD4+CD45RA+PD1+.

Disorders in the immune system happening due to aging or chronic inflammatory diseases have been defined as immunosenescence and are distinct from the dysfunction that occurs following chronic infections, demarked as immunoexhaustion [16]. Immunosenescent alterations affect both the innate and acquired immune systems, leading to a plethora of changes in dendritic cells, macrophages, neutrophils, and lymphocytes [17]. They lead to inflammatory cytokine production, globally characterized as senescence-associated secretory phenotype (SASP), and predispose to age-related diseases, such as cardiovascular, neuro-generative, autoimmune, and malignant diseases and defective control of unrelenting infections [18,19]. Several phenotypic alterations of senescent lymphocytes have been well described, including reduced length and activity of telomeres and alterations of membrane receptors. In the present study we selected the presence of CD31, CCR7, and CD28 molecules as markers of naïve or early differentiated lymphocytes and the reduction or elimination of CD28 co-stimulatory molecules, the increased expression of CD57, and the re-expression of CD45RA molecules on effector memory T lymphocytes as markers of advanced and/or terminally differentiated cells. As for the B lymphocytes, the expression of IgD and CD27 molecules were assessed, with the presence of CD27 signalizing advanced differentiated B lymphocyte subtypes.

Senescent lymphocytes are highly cytotoxic and inflammatory cells, characterized by detrimental atherogenic and carcinogenic functions [20]. On the other hand, exhausted lymphocytes are derived as a result of chronic infections or inflammation, express the PD1 molecule, and are anergic but not cytotoxic [16,21,22]. In CKD, the observed changes in the immune system resemble those of aging or chronic inflammation, although the latest data, apart from similarities, point to certain differences and indicate a unique lymphocyte phenotype, distinctive for CKD. The senescence prompted by CKD is mostly influenced by local tissue alterations, while senescent cell effector molecules, such as SASP, grant local and systemic modifications in the dysregulation of the immune system. As well, uremia further increases this reply by the disturbance of immune cell operation. Reduced numbers of naïve lymphocytes, regulatory T and dendritic cells, and increased exhausted lymphocytes are the main characteristics [23]. Throughout renal injury and CKD supervenes metabolic reprogramming, which is interceded by hypoxia inducible factor-1α (HIF-1α). This can cause greater glycolysis and changes in amino acid metabolism in immune system cells. Furthermore, the stimulation of innate recognition receptors, including Toll-like ones (TLRs), NOD-like ones (NLRs), and inflammasomes, prompts paths within cells that link up on nuclear factor κB (NF-ĸB). As a consequence, pro-inflammatory cytokines [namely, tumor necrosis factor (TNF) andinterleukin-1β (IL-1β)] and chemokines are produced, generating a positive feedback mechanism that maintains the inflammatory response [24,25]. Patients with CKD or KF display reduced naïve T and B cells, premature thymic dysfunction, and raised homeostatic proliferation of naive T cells. In addition to a decreased thymic T cell output, they have shorter telomeres in CD4+ and CD8+ T cells [26,27]. The proportion of naïve and regulatory T lymphocytes is reduced in CKD. These effects were more prominent in hemodialysis, as compared with peritoneal dialysis patients [1,28,29]. The beneficial effect of peritoneal dialysis was mainly attributed to residual kidney function and the preservation of urine output. The maintenance of urine output in dialysis patients, even at very low levels, is crucial as it contributes to optimal removal of PBUTs or middle molecules acting as uremic toxins, which are inefficiently removed by dialysis [30]. In the present study, we found a significant impact of urine output in HA and IxS blood levels. However, in our study, the PBUT levels in patients on HD did not have statistically significant correlations with the method of dialysis or membrane materials. In another study, it was found that dialysis membranes can have impacts on the levels of perfluorochemicals and could be helpful for patients [31].

Very interestingly, a multivariate analysis, including age, residual kidney function (defined as urine output ≥ 500 mL/d), and PBUT levels as independent factors, to assess lymphocyte alterations, showed that the age and IxS were the main independent parameters implicated in the reduction inCD4 and B lymphocytes and their naïve and early differentiated subsets, while pCS levels were the main factor correlated with exhausted CD4+PD1+ cells. PBUTs have several detrimental effects on the immune system. Indeed, the increased concentrations of the two most important solutes, pCS and IxS, have been associated with the adaptive immunity deficiencies common in CKD. As CKD is characterized by increased production and accumulation of PBUTs, which are exclusively produced by gut microbiota during protein fermentation, it seems reasonable that these molecules may act as the link between the gut microbiota and certain immune deficiencies in CKD [32]. Indeed, various CKD complications have been attributed to PBUT accumulation, such as cardiovascular diseases, anemia, and mineral–bone disorders. A systematic review of 27 studies confirms that IxS and pCS have important roles in vascular and kidney disease progression [33].

The exact mechanisms by which PBUTs can affect immune reactions are not completely defined. However, in vitro and in vivo experimental evidence has shown that pCS and IxS may cause significant deregulation of dendritic cells, leading to impaired phagocytosis, antigen processing, and presentation to lymphocytes. Moreover, administration of pCS in a mouse model remarkably reduced B lymphocytes, mainly affecting their naïve and memory subtypes [11]. A recent study demonstrates the association of IxS levels with lymphopenia and an increased neutrophil/lymphocyte ratio [34]. Although the association between increased PBUT and impairment in adaptive immunity has been sporadically described, their possible correlation with certain lymphocyte alterations in KF and the shift to immunosenescent and immunoexhausted phenotypes have not been investigated before. Even more, the close associations of HA and IxS with immunosenescence and pCG with immunoexhaustion have not been described in humans.

Hippuric acid, produced by the conjugation of glycine and benzoic acid, derives from the microbial degradation of polyphenolic dietary compounds found in plant-based foods, fruits, vegetables, tea, and coffee. The kinetics of HA are rather complicated as its levels are increased by dietary habits (fruit and vegetable consumption) and reduced during aging. The urinary excretion of HA is increased with age and age-related diseases, such as cognitive impairments, rheumatic diseases, sarcopenia, and hypomobility, and has been proposed as a potential hallmark of aging, frailty, and age-related diseases [35,36]. There are currently not enough data regarding the HA concentration and kinetics in CKD; its protein binding is reduced to 37% in uremic conditions. It is excreted in the urine (almost 400 mg per day), and apparently, in KF its levels are strongly influenced by reduced urine output [9]. Therefore, HA blood levels were increased in our patients on HD, despite restrictions in fruit and vegetable consumption.

IxS is a small substance with more than 90% protein binding, derived from the breakdown of tryptophan by intestinal microbiota. In healthy individuals, kidneys can succeed to eliminate IxS levels by tubular secretion, something that cannot be achieved in KF [37]. IxS proved to be the most important PBUT in our study, acting as an independent factor for senescent phenotypic alterations of lymphocytes. A recent experiment showed that the incubation of proximal tubular epithelial cells in the presence of IxS leads to the upregulation in the SASP factors, accelerates the cellular senescence of epithelial cells, and finally promotes kidney fibrosis via TNF-α, NF-ĸB signaling pathways, and the epithelial–mesenchymal transition process [38]. No clinical studies have proven these results, but it seems extremely promising that IxS may be implicated in the senescent progression of other cell types, a hypothesis that has to be investigated.

One of the novel findings of this study was the significant correlation of pCS levels with exhausted CD4 lymphocytes. The accumulation of pCS, even at early stages of CKD, is associated with systemic toxic effects, in addition to further deterioration of kidney function [39]. In vitro experiments have shown that pCS can down-regulate interleukin (IL)-7 production and inhibit the proliferation of B lymphocytes, by their accumulation in the G1 phase. In vivo studies have proven a detrimental effect of high blood pCS concentrations on peripheral B lymphocytes in mice with renal dysfunction [40]. Oxidative stress and inflammation are directly promoted by both IxS and pCS, through the activation of inflammatory cytokines and coagulation pathways, and lead to protein energy wasting syndrome and vascular calcification [41,42,43,44].

This study has various limitations. A major one is the small number of selected patients and healthy individuals. Furthermore, we excluded patients with possible alterations in the immunological profile, such as those with diabetes mellitus and/or autoimmune disease. Consequently, this study group cannot be representative of the whole population on HD. Another limitation is the possible inclusion of patients with chronic glomerulous disease that may not have been discovered with a clinical diagnosis or tissue biopsy. Moreover, we examined only the above uremic toxins. In future research, the inclusion of other uremic bound toxins, such as acrolein and persisted organic pollutants, could be useful [45].

The close correlation of the PBUT levels with certain phenotypic changes of lymphocytes in patients on HD has not been described before and seems to reveal a new aspect of the pathogenesis in immunological consequences in CKD. However, in the present cross sectional study, we can only describe the correlations between uremic toxin levels and lymphocyte subpopulations and suggest a possible role of PBUTs in the alterations of immune profile, leading to well-defined clinical consequences.

## 5. Conclusions

In conclusion, we described an increase in protein-bound uremic toxins in patients on hemodialysis strongly associated with the immunosenescent and immunoexhausted phenotype of these patients. PBUTs seem to be the link between uremic dysbiosis and deficiencies of the adaptive immunity in chronic kidney disease; specifically, HA and IxS were associated with an immunosenescent phenotype, while pCS was associated with the immune-exhausted lymphocyte phenotype.

## Figures and Tables

**Table 1:** Demographics, characteristics, and laboratory findings in the whole cohort of patients and in two subgroups, those with or without RKF (residual urinary output ≥500 mL/24 h). P values are referred to differences between patients subgroups.

Parameters	All Patients	RKF *		*p*
		Yes	No	
n	54	21	33	
Age (years)	51.3 ± 16.9	51 ± 15.9	51.4 ± 17.7	0.922
Sex (female/male)	23/31	10/11	13/20	0.551
Dialysis vintage (months)	67 (20.7–95.2)	21 (9.5–57.5)	85 (49.5–111.5)	<0.001
BMI * (kg/m^2^)	24.5 ± 3.6	24.7 ± 4.4	24.4 ± 3.1	0.551
HD * (%)	30 (55.5)	14 (66.6)	16 (48.4)	0.19
HDF * (%)	24 (44.4)	7 (33.3)	17 (51.5)	0.19
Comorbidity				
Arterial hypertension (%)	36 (66.7)	14 (66.7)	22 (66.7)	0.742
Dyslipidemia (%)	17 (31.5)	9 (42.9)	8 (24.2)	0.198
Primary cause of CKD *				
Primary glomerulonephritis (%)	16 (29.6)	6 (28.6)	10 (30.3)	0.891
Obstructive uropathy (%)	9 (16.7)	3 (14.3)	6 (18.2)	0.708
ADPKD * (%)	7 (13)	2 (9.5)	5 (15.2)	0.548
Hypertension (%)	3 (5.6)	1 (4.8)	2 (6.1)	0.839
Alport syndrome (%)	2 (3.7)	1 (4.8)	1 (3)	0.742
Other (%)	2 (3.7)	0 (0)	2 (6.1)	0.785
Unknown (%)	15 (27.8)	8 (38.1)	7 (21.2)	0.176
Medication				
Epoetin (iu/week)	4500 (2000–8000)	5250 (2625–8000)	4000 (0–8000)	0.740
Iron (mg/month)	200 (100–200)	200 (150–300)	100 (0–200)	0.122
Paricalcitol (mg/week)	0 (0–5)	0 (0–5)	0 (0–7.5)	0.598
Laboratory parameters				
WBC * (cells/μL)	7100 (5500–8325)	6900 (5450–7800)	7100 (5600–8500)	0.619
Neutrophils (cells/μL)	4550 (3475–5500)	4500 (3400–5200)	4600 (3750–5850)	0.267
Lymphocytes (cells/μL)	1400 (1175–1800)	1600 (1300–1900)	1400 (1000–1750)	0.105
Monocytes (cells/μL)	602.4 (538.2–758.4)	585.2 (415.1–661.8)	641.7 (543.8–777.6)	0.189
Hematocrit (%)	36.1 ± 3.1	35.9 ± 2.6	36.2 ± 3.4	0.729
Hemoglobin (g/dL)	11.8 ± 0.9	11.8 ± 0.8	11.8 ± 1.0	0.950
Platelets (10^3^/μL)	227 (184.2–265)	215 (169–263.5)	228 (200.5–268)	0.312
Urea (mg/dL)	127 (111.5–151.5)	125 (107.7–154.5)	129 (114.2–149.5)	0.677
Creatinine (mg/dL)	9.4 (7.3–10.9)	8.3 (5.7–9.6)	10.4 (8.3–11.9)	0.005
Calcium (mg/dL)	9.1 (8.8–9.3)	9.1 (8.9–9.2)	9.2 (8.8–9.4)	0.758
Phosphorus (mg/dL)	4.3 (3.7–5)	4.3 (3.7–5)	4.3 (3.6–4.9)	0.929
Parathyroid hormone (pg/mL)	206.5 (106.2–370.7)	180 (119–330)	212 (95.5–378.5)	0.825
Cholesterol (mg/dL)	156.5 (122.5–172.5)	164 (128.2–183.7)	152.5 (116.2–168.2)	0.244
Triglycerides (mg/dL)	129 (84.7–163.7)	127 (82–180)	131 (92.5–161)	0.768
HDL * (mg/dL)	42.5 (35.7–50.5)	42 (31.2–49.7)	43 (36.7–52)	0.5
LDL * (mg/dL)	78 (62.2–94.5)	84 (76–103)	75 (57.5–87.5)	0.056
Ferritin (ng/mL)	301 (158–440)	322 (200–440)	296 (110.2–461.5)	0.905
Albumin (g/dL)	4.1 (3.9–4.3)	4.15 (3.9–4.4)	4.1 (3.9–4.3)	0.491
LDH * (IU/L)	164 (145–187.2)	152 (141–178)	169 (148–195)	0.148
C-reactive protein (mg/L)	2.3 (1.4–4.2)	2.4 (1.4–4)	2.1 (1.4–5.2)	0.905

* Residual kidney function (RKF); body mass index (BMI); hemodialysis (HD); hemodiafiltration (HDF); chronic kidney disease (CKD); autosomal dominant polycystic kidney disease (ADPKD); white blood cells (WBC); high-density lipoprotein (HDL); low-density lipoprotein (LDL); lactate dehydrogenase (LDH).

**Table 2:** Differences in absolute number and percentage (%) in complete blood count parameters between control group and patients.

Parameters	Control Group	All Patients	*p*
n	31	54	
Age (years)	51.3 ± 17.2	51.3 ± 16.9	0.993
Sex (female/male)	15/16	23/31	0.605
Laboratory parameters			
WBC (cells/μL)	6200 (5300–7100)	7100 (5500–8325)	0.046
Neutrophils (cells/μL)	3400 (2700–4200)	4550 (3475–5500)	0.001
Lymphocytes (cells/μL)	2100 (1600–2500)	1400 (1175–1800)	<0.001
Monocytes (cells/μL)	510.4 (450.8–583.2)	602.4 (538.2–758.4)	0.001
Hematocrit (%)	40.7 ± 3.1	36.1 ± 3.1	<0.001
Hemoglobin (g/dL)	13.7 ± 1.1	11.8 ± 0.9	<0.001
Platelets (10^3^/μL)	216 (199–245)	227 (184.2–265)	0.729
CD4 (%)	51.1 (44.3–57.1)	47.05 (42–53.7)	0.074
CD4 (cells/μL)	999 (786–1237)	679.5 (483–862.2)	<0.001
CD8 (%)	21.2 (17–31.4)	27.2 (19.3–34.7)	0.131
CD8 (cells/μL)	451 (296–746)	377 (261.7–531.5)	0.130
CD4/CD8	2.229 (1.4–3.3)	1.75 (1.3–2.5)	0.074

**Table 3:** Differences in absolute number and percentage (%) of T lymphocyte subpopulations according to their differentiation in patients on hemodialysis (HD) versus control.

T Lymphocyte Subpopulations	Control Group	HD Patients	*p*
N	31	54	
Early differentiated cells			
CD4+CD45RA+CD31+ (cells/μL)	250 (137–352)	128.5 (97.7–212)	0.001
CD4+CD45RA+CCR7+ (cells/μL)	320 (219–484)	241.5 (158.7–327.7)	0.042
CD4+CD28+CD57− (cells/μL)	964 (740–1178)	592 (413.7–751)	<0.001
CD4+CD45RA+CD57− (cells/μL)	401 (225–541)	251 (152.5–344.5)	<0.001
CD8+CD45RA+CD31+ (cells/μL)	164 (32–220)	114.5 (47–184.7)	0.471
CD8+CD45RA+CCR7+ (cells/μL)	121 (22–164)	98.5 (33.5–179.5)	0.596
CD8+CD28+CD57− (cells/μL)	263 (188–371)	160 (111.5–210.7)	<0.001
CD8+CD45RA+CD57− (cells/μL)	108 (39–192)	81 (43–122)	0.33
Memory cells			
CD4+CD45RA−CCR7+ (CM) * (cells/μL)	563 (353–701)	345.5 (262.5–498.7)	0.01
CD4+CD45RA−CCR7− (EM)* (cells/μL)	8 (1–15)	5 (2–11.7)	0.876
CD8+CD45RA−CCR7+ (CM)* (cells/μL)	144 (30–295)	113.5 (44.7–333.7)	0.805
CD8+CD45RA−CCR7− (EM)* (cells/μL)	28 (2–89)	13 (5–32.2)	0.193
Advanced differentiated cells			
CD4+CD45RA+CCR7− (EMRA) * (cells/μL)	12 (3–37)	14 (5.7–23.7)	0.739
CD4+CD45RA−CD57+ (cells/μL)	14 (4–26)	13 (5–30)	0.739
CD4+CD28+CD57+ (cells/μL)	7 (3–12)	5 (2–10)	0.204
CD8+CD45RA+CCR7− (EMRA) * (cells/μL)	52 (3–152)	23.5 (4–83.5)	0.25
CD8+CD45RA−CD57+ (cells/μL)	55 (11–98)	36 (14.7–107.2)	0.852
CD8+CD28+CD57+ (cells/μL)	9 (4–15)	7 (4–13)	0.791
Terminally differentiated cells			
CD4+CD45RA+CCR7−CD28− (cells/μL)	2 (0–10)	2 (1–6)	0.908
CD4+CD28−CD57+ (cells/μL)	18 (3–42)	19.5 (5.7–48)	0.641
CD8+CD45RA+CCR7−CD28− (cells/μL)	36 (3–65)	11 (2–49.2)	0.204
CD8+CD28−CD57+ (cells/μL)	73 (32–245)	98.5 (32.5–207.2)	0.996

* Central memory (CM); effector memory (EM); effector memory expressing CD45RA (EMRA).

**Table 4:** Differences in absolute number and percentage (%) in B lymphocytes in patients on hemodialysis (HD) versus control.

B Lymphocyte Subpopulations	Control Group	HD Patients	*p*
n	31	54	
CD19 (%)	14.7 (9.5–16.3)	6.4 (4.5–8.5)	<0.001
CD19 (cells/μL)	248 (163–388)	91 (52.2–131.2)	<0.001
CD19+IgD+CD27− (cells/μL)	144 (91–258)	56.5 (27.7–96.2)	<0.001
CD19+IgD+CD27+ (cells/μL)	23 (11–32)	5 (3–10.2)	<0.001
CD19+IgD−CD27+ (cells/μL)	35 (22–61)	13 (7.7–18.5)	<0.001
CD19+IgD−CD27− (cells/μL)	26 (14–43)	7 (4.7–12)	<0.001

**Table 5:** Differences in absolute number and percentage (%) in exhausted T lymphocytes in patients of hemodialysis (HD) versus control.

Exhausted T Lymphocyte Subpopulations	Control Group	HD Patients	*p*
n	31	54	
CD4+PD1+ (%)	7.7 (5.3–11.7)	11.7 (7–17.8)	0.009
CD4+PD1+ (cells/μL)	68 (52–117)	80 (42.5–120.2)	0.982
CD4+CD45RA−PD1+ (%)	6.7 (3.9–10.5)	10.9 (5.8–14.8)	0.005
CD4+CD45RA−PD1+ (cells/μL)	54 (33–110)	68 (36–98.7)	0.77
CD4+CD45RA+PD1+ (%)	1.2 (0.6–2)	1.2 (0.6–1.8)	0.722
CD4+CD45RA+PD1+ (cells/μL)	12 (5–21)	8 (3.7–14.2)	0.099
CD8+PD1+ (%)	32.4 (18.1–45.5)	28.7 (11.3–46.2)	0.335
CD8+PD1+ (cells/μL)	125 (91–212)	75 (39.2–176)	0.066
CD8+CD45RA−PD1+ (%)	20.5 (9.9–32)	13.1 (6.6–35.5)	0.291
CD8+CD45RA−PD1+ (cells/μL)	87 (41–171)	38.5 (20–117)	0.088
CD8+CD45RA+PD1+ (%)	7.9 (3.1–16.3)	5.9 (1.7–17.4)	0.612
CD8+CD45RA+PD1+ (cells/μL)	49 (11–86)	27 (4.7–70.2)	0.146

**Table 6:** Correlation of uremic toxins with T cells.

	Total HA *		Free HA *		Total IxS *		Free IxS *		Total pCS *		Free pCS *	
	r	*p*	r	*p*	r	*p*	r	*p*	r	*p*	r	*p*
Total lymphocytes	−0.3	0.01	−0.3	0.02	−0.2	0.1	−0.3	0.017	0.05	0.71	−0.1	0.31
CD4 cells	−0.3	0.02	−0.3	0.01	−0.3	0.02	−0.4	0.005	−0.1	0.4	−0.1	0.7
Early differentiated												
CD4+C45RA+CD31+	−0.3	0.037	−0.3	0.027	−0.1	0.2	−0.1	0.1	−0.2	0.08	−0.1	0.9
CD4+CD45RA+CCR7+	−0.25	0.06	−0.27	0.04	−0.2	0.1	−0.2	0.08	−0.17	0.2	−0.1	0.9
CD4+CD45RA+CD57−	−0.3	0.03	−0.3	0.02	−0.3	0.02	−0.3	0.01	−0.1	0.3	−0.2	0.8
CD4+CD28+CD57−	−0.3	0.05	−0.3	0.03	−0.3	0.01	−0.3	0.009	−0.1	0.5	−0.1	0.6
Memory												
CD4+CD45RA−CCR7+ (CM) *	−0.27	0.09	−0.24	0.07	−0.1	0.6	−0.16	0.2	−0.1	0.5	−0.1	0.5
CD4+CD45RA−CCR7− (EM) *	0.02	0.9	−0.02	0.9	−0.02	0.9	−0.03	0.8	−0.04	0.7	−0.07	0.5
Advanced differentiated												
CD4+CD45RA+CCR7− (EMRA) *	−0.2	0.1	−0.2	0.08	−0.1	0.6	−0.1	0.2	−0.1	0.4	−0.1	0.3
CD4+CD45RA−CD57+	0.2	0.2	0.2	0.2	0.2	0.09	0.2	0.2	0.1	0.7	0.1	0.1
CD4+CD28+CD57+	0.1	0.2	0.1	0.2	0.1	0.2	0.07	0.5	0.04	0.7	0.1	0.4
CD4+CD28−CD57−	0.2	0.1	0.2	0.1	0.3	0.04	0.2	0.2	0.3	0.01	0.3	0.04
Terminally differentiated												
CD4+CD45RA+CCR7−CD28− (EMRA/CD28−)	−0.4	0.008	−0.3	0.01	−0.04	0.7	−0.2	0.1	−0.01	0.8	0.01	0.5
CD4+CD28−CD57+	0.2	0.8	0.01	0.9	0.1	0.2	0.1	0.4	0.1	0.4	0.2	0.08

* Hippuric acid (HA); indoxyl sulfate (IxS); p-cresyl sulfate (pCS); central memory (CM); effector memory (EM); effector memory expressing CD45RA (EMRA).

**Table 7:** Correlation of uremic toxins with exhausted T cells.

	Total HA *		Free HA *		Total IxS *		Free IxS *		Total pCS *		Free pCS *	
	R	*p*	r	*p*	r	*p*	r	*p*	r	*p*	r	*p*
CD4+PD1+	−0.06	0.6	−0.04	0.2	0.1	0.3	0.07	0.58	0.3	0.02	0.3	0.01
CD4+CD45RA+PD1+	−0.1	0.1	−0.1	0.9	0.1	0.43	0.1	0.63	0.3	0.039	0.3	0.045
CD4+CD45RA−PD1+	−0.06	0.9	0.1	0.7	0.2	0.2	0.1	0.48	0.2	0.1	0.2	0.06

* Hippuric acid (HA); indoxyl sulfate (IxS); p-cresyl sulfate (pCS).

**Table 8:** Correlation of uremic toxins with B cells.

	Total HA *		Free HA *		Total IxS *		Free IxS *		Total pCS *		Free pCS *		Total pCG *		Free pCG *		CMPF *	
	r	*p*	r	*P*	r	*p*	r	*p*	r	*p*	r	*p*	r	*p*	r	*p*	r	*p*
CD19	−0.2	0.08	−0.2	0.05	−0.2	0.07	−0.4	0.008	0.1	0.4	0.1	0.4	−0.1	0.51	−0.1	0.516	−0.1	0.206
CD19+IgD+CD27−	−0.3	0.04	−0.3	0.03	−0.3	0.07	−0.3	0.01	0.1	0.6	−0.1	0.3	−0.1	0.41	−0.1	0.437	−0.2	0.047
CD19+IgD+CD27+	−0.1	0.1	−0.2	0.1	−0.2	0.09	−0.3	0.01	−0.2	0.1	−0.2	0.1	−0.3	0.014	−0.3	0.015	−0.1	0.731
CD19+IgD−CD27+	−0.2	0.2	−0.2	0.1	−0.1	0.2	−0.2	0.07	0.1	0.9	−0.1	0.2	−0.1	0.319	−0.1	0.3	−0.1	0.828
CD19+IgD−CD27−	−0.1	0.2	−0.1	0.2	−0.1	0.7	−0.2	0.2	0.1	0.9	−0.1	0.2	−0.1	0.162	−0.2	0.146	−0.1	0.63

* Hippuric acid (HA); indoxyl sulfate (IxS); p-cresyl sulfate (pCS); p-cresyl glucuronide (pCG); 3-carboxy-4-methyl-propyl-2-furanpropanoic acid (CMPF).

**Table 9:** Independent factors that were associated and could predict peripheral CD4 and B lymphocyte subpopulations.

	R^2^	Adjusted R^2^	B Coefficient	Confidence Interval (Lower–Upper)		*p*
CD4 cells						
Free IxS *	0.20	0.16	−855.63	−1481	−220.17	0.007
Age			−4.61	−9.1	−0.16	0.04
CD4CD31+ cells						
Age	0.21	0.20	−3	−4.6	−1.3	0.001
Naïve CD4 cells						
Free IxS *	0.18	0.13	−410	−813	−7.44	0.04
CD4CD45RA+CD57−						
Free IxS *	0.22	0.19	−415.9	−763.4	−68.4	0.02
Age			−3.4	−5.9	−0.9	0.009
CD4+PD1+						
Total pCS *	0.28	0.19	−2.9	0.72	50.14	0.04
B lymphocytes						
Free IxS *	0.38	0.35	−208.9	−322.78	−75.42	0.001
Age			−1.6	−2.4	−0.8	<0.0001
CD19IgD+CD27−						
Free IxS *	0.31	0.28	−137.6	−228	−47.2	0.004
Age			−1.1	−1.8	−0.5	0.001
CD19IgD+CD27+						
Free pCG *	0.29	0.25	−10.5	−20.3	−0.8	0.03

* Indoxyl sulfate (IxS); p-cresyl sulfate (pCS); p-cresyl glucuronide (pCG).

## Data Availability

The datasets used and analyzed during the current study are available from the corresponding author on reasonable request.

## Supplementary Materials

The following supporting information can be downloaded athttps://www.mdpi.com/article/10.3390/biomedicines11092504/s1: Figure S1: Gating strategy for CD4 and CD8 cells and their subsets, based on the presence of CD45RA, CCR7, CD31, CD57, and CD28; Figure S2: Gating strategy for CD19+ cells and their subsets based on the presence of IgD and CD27.
