# Supplementary figures and images for: Immunosenescence and Immune Exhaustion Are Associated with Levels of Protein-Bound Uremic Toxins in Patients on Hemodialysis
Source: Biomedicines. 2023 Sep 11;11(9):2504. doi: 10.3390/biomedicines11092504 (PMC10525954; doi:10.3390/biomedicines11092504)

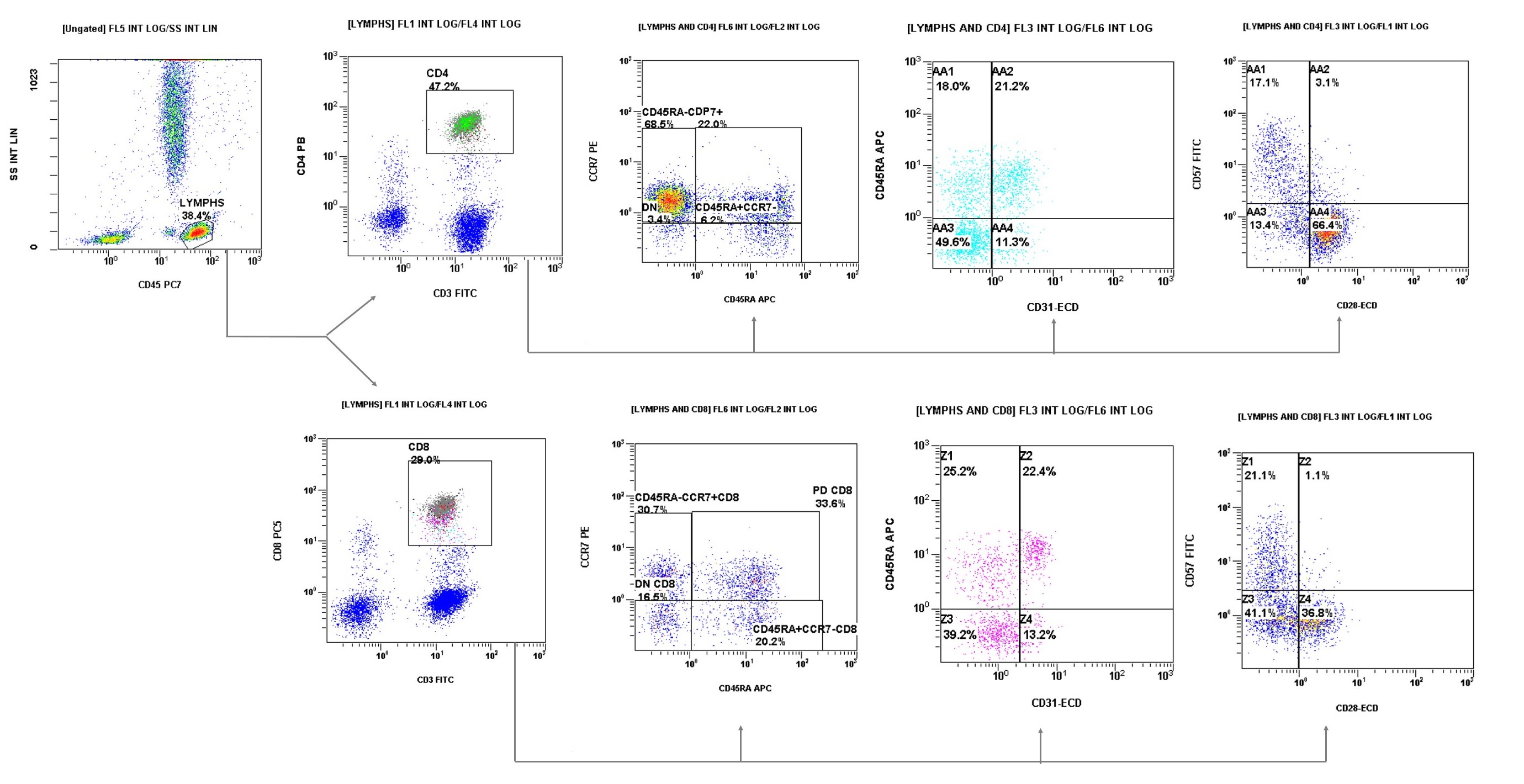

Supplement: Supplementary file 1 [file biomedicines-11-02504-s001.zip › Figure S1.jpg]

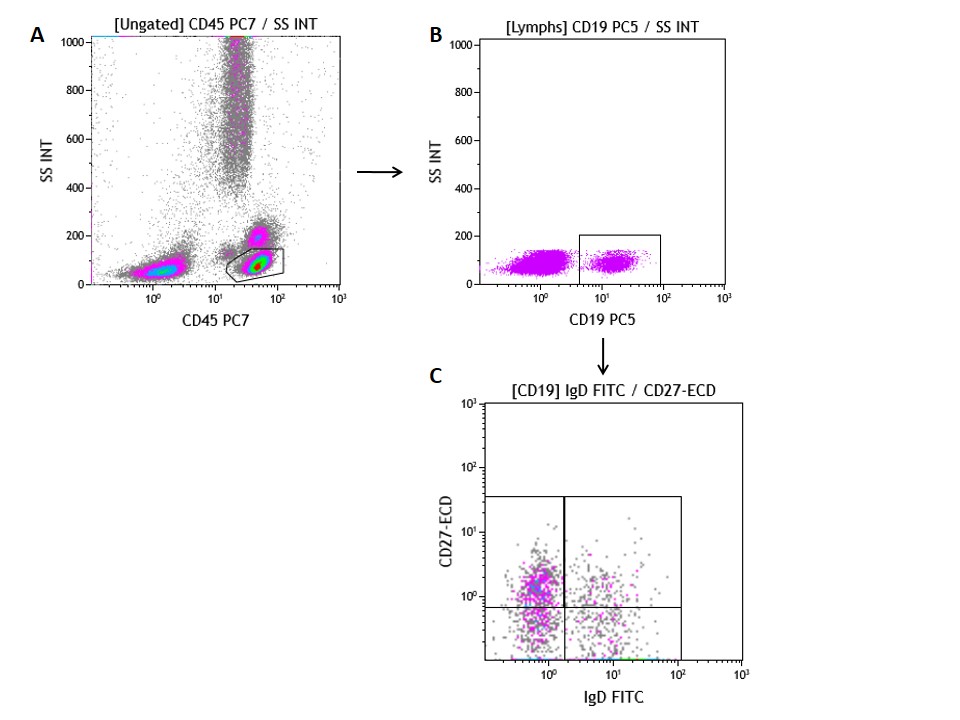

Supplement: Supplementary file 1 [file biomedicines-11-02504-s001.zip › Figure S2.jpg]
